# Results of the Dyslipidemia International Study (DYSIS)-Middle East: Clinical Perspective on the Prevalence and Characteristics of Lipid Abnormalities in the Setting of Chronic Statin Treatment

**DOI:** 10.1371/journal.pone.0084350

**Published:** 2014-01-06

**Authors:** Saud N. Al Sifri, Wael Almahmeed, Sami Azar, Osama Okkeh, Peter Bramlage, Claus Jünger, Islam Halawa, Baishali Ambegaonkar, Sameh Wajih, Philippe Brudi

**Affiliations:** 1 Endocrinology Department, Al Hada Military Hospitals, Taif, Saudi Arabia; 2 Institute of Cardiac Sciences, Sheikh Khalifa Medical City, Abu Dhabi, United Arab Emirates; 3 American University of Beirut Medical Center, Beirut, Lebanon; 4 Istishari Hospital, Amman, Jordan; 5 Institut für Pharmakologie und präventive Medizin, Mahlow, Germany; 6 Institut für Herzinfarktforschung, Ludwigshafen, Germany; 7 University Medical Center, Johannes Gutenberg University, Mainz, Germany; 8 Medical Department, MSD Saudi Arabia, Riyadh, Saudi Arabia; 9 Merck & Co., Inc., Whitehouse Station, New Jersey, United States of America; 10 Medical Department, MSD United Arab Emirates, Abu Dhabi, United Arab Emirates; Medical University Innsbruck, Austria

## Abstract

**Background:**

Therapeutic intervention with low-density lipoprotein cholesterol-lowering agents known as statins has been demonstrated to reduce cardiovascular risk. However, many patients on statin treatment have persistent dyslipidemia and remain at a high risk of cardiovascular disease. Therefore, the objective of this study was to assess the frequency of lipid abnormalities in patients receiving chronic statin treatment.

**Methods:**

As part of an international, cross-sectional, observational study, DYSIS-Middle East enrolled 2,182 patients in the United Arab Emirates (UAE), Saudi Arabia, Lebanon and Jordan. All patients were over 45 years of age and had been on statin treatment for at least three months. Data on demographics, lipid parameters and cardiovascular risk profile were recorded. Cardiovascular risk was defined according the guidelines of the European Society of Cardiology.

**Results:**

The majority of patients (82.6%) were classified as being at very high risk of cardiovascular events, and 61.8% of all patients did not attain LDL-C target levels. Low high-density lipoprotein cholesterol levels and elevated triglyceride levels were noted in 55.5% and 48.5% of patients, respectively. Multivariate logistical regression modeling indicated that factors independently associated with LDL-C levels not being at goal were lifestyle choices, diabetes mellitus, ischemic heart disease, and blood pressure ≥ 140/90 mmHg.

**Conclusions:**

Almost two-thirds of statin-treated patients in the United Arab Emirates, Saudi Arabia, Lebanon and Jordan had inadequately controlled lipid levels. More comprehensive surveillance, awareness and treatment regimens, as well as modification of lifestyle choices, is necessary to halt the rise in cardiovascular disease-related mortality.

## Introduction

Cardiovascular disease (CVD), which encompasses conditions such as coronary heart disease, stroke, peripheral artery disease, is the leading cause of mortality worldwide. Estimates from the World Health Organization suggest that in 2008, approximately 17.3 million individuals died from CVD, corresponding to 30% of global deaths. By 2030, almost 25 million individuals are expected to die from CVD, primarily heart disease and stroke [1]. In the Middle East, the rate of increase in CVD-associated mortalities is one of highest in the world [2]. Data from 2008 indicate that CVD accounted for almost half of all deaths in Saudi Arabia and Lebanon (42 and 45%, respectively); however, the accuracy of these figures is uncertain because neither country has a respective disease register [3,4].

Risk factors for CVD are well documented and include lifestyle choices such as smoking and a sedentary lifestyle, and metabolic disorders such as obesity, diabetes and dyslipidemia. Meta-analyses of large, randomized studies have demonstrated that modification of these risk factors, in particular by lowering low-density lipoprotein cholesterol (LDL-C) levels with statins (HMG-CoA reductase inhibitors), reduces the likelihood of CVD-related morbidity and mortality [5-8]. In addition to elevated LDL-C levels, high triglyceride levels and low high-density lipoprotein cholesterol (HDL-C) levels may be important contributors to the residual risk of CVD [9,10].

Despite pharmacological intervention and encouragement to make lifestyle changes, many patients do not achieve target lipid levels and a considerable risk of CVD remains [9]. For example, in the European Action on Secondary and Primary Prevention through Intervention to Reduce Events (EUROASPIRE) II study, lipid-lowering agents were being taken by 61% of patients, but 58% had above-target total cholesterol levels [11,12]. Although the use of lipid-lowering agents had increased to 89% of patients in the more recent EUROASPIRE III survey, 46% had elevated total cholesterol levels and almost two-fifths of treated patients did not reach target total cholesterol levels [11,13]. In EUROASPIRE IV (presented at the European Society of Cardiology Meeting 2013), 87% of patients were taking lipid lowering drugs (almost exclusively statins). Of these, 58% had a LDL-C level of < 2.5 mmol/L (100 mg/dL) but only 21% reached an LDL-C target of <1.8mmol/L (70mg/dL). The European Society of Cardiology (ESC) published up-to-date recommendations for the prevention and treatment of CVD [14].

The Dyslipidemia International Study (DYSIS) was a cross-sectional, observational study that assessed lipid abnormalities in the setting of chronic statin treatment. The objectives of the study were: (i) to assess the prevalence of lipid abnormalities in patients receiving chronic statin treatment; (ii) to better characterize the key risk factors for CVD; and, (iii) to use the data obtained to inform and improve clinical practice. Results from the Canadian and European DYSIS cohorts, which included over 22,000 patients, have been previously reported [15]. Here, findings from DYSIS-Middle East, conducted in the United Arab Emirates (UAE), Saudi Arabia, Lebanon and Jordan, are presented.

## Methods

### Study design and patients

DYSIS-Middle East was an epidemiologic, observational, cross-sectional, multicenter study conducted in the UAE (n=300), Saudi Arabia (n=1265), Lebanon (n=395) and Jordan (n=222). Data were collected between December 2011 and April 2012 in local-language case report forms and held at the Institut für Herzinfarktforschung Ludwigshafen, Germany. Centers (n=142) selected were office based cardiologists that were distributed throughout participating countries. Prior to study initiation, on the 16^th^ of November 2011, the Sheikh Khalifa Medical City Research Ethics Committee approved the study protocol and patients’ written informed consent was obtained. A total of 2,182 patients were enrolled, all aged over 45 years, receiving statin treatment for at least three months and having at least one fasting blood lipid profile available while on statins. Only the fasting lipid profile was taken as the basis for the further calculations.

### Data collection

Data on age, sex, ethnicity, family history of CVD, tobacco smoking, blood pressure, waist circumference, body mass index (BMI), and presence of CVD, diabetes mellitus and metabolic syndrome were recorded. Lipid levels (LDL-C, HDL-C and triglycerides) were obtained from the most recent blood sample taken within the previous six to 12 months. For diabetic patients, HbA1c and fasting plasma glucose levels were also analyzed. Information on the name and daily dose of statin at the time of the most recent blood sample, as well as other lipid-modifying treatments, was documented. Statin dose level was normalized using a potency calculation, and the potency of different statins was benchmarked against six simvastatin dose levels (5, 10, 20, 40, 80 and 160 mg/day), with potency scores ranging from 1 (5 mg/day simvastatin) to 6 (160 mg/day simvastatin) [16,17].

### Lipid level determination

The DYSIS protocol was modified in DYSIS Middle East to make use of the CardioChek^®^ device (http://www.cardiocheck.com). The purpose was to reliably collect lipid measurements. The provided LDL-C test strip measures LDL-C across a range of 50 to 200 mg/dL (1.29-5.18 mmol/L) in about 2 minutes. A linearity study covering the range of 48-244 mg/dL (1.24-6.32 mmol/L) had a correlation coefficient of 0.973 with the regression line of y = 0.8909x + 13.24, with an average recovery of 103.3%. The performance of this determination was previously determined in a total of 60 untrained users. In these tests 98.3% of LDL-C determinations were in the range of ± 15% (59/60) (Direct LDL Package Insert at http://www.cardiocheck.com).

### Cardiovascular risk classification

Cardiovascular risk and abnormalities in LDL-C, HDL-C and triglyceride levels were defined according to the ESC guidelines (2011) [18]. LDL-C level treatment goals were < 3.0 mmol/L in patients with SCORE risk 1-4%, < 2.5 mmol/L in patients with SCORE risk 5-9%, < 1.8 mmol/L in patients with CVD, DM, and/or SCORE risk ≥10%. Variables independently associated with dyslipidemia were assessed using multivariate logistic regression analysis, with the following variables considered: age, sex, first-grade family history of premature CVD, current smoker, sedentary lifestyle, alcohol consumption (> 2 units/week), BMI ≥ 30 kg/m^2^ (i.e., obesity), waist circumference (> 102 cm in men, > 88 cm in women), history of hypertension, diabetes mellitus, ischemic heart disease, cerebrovascular disease, heart failure, peripheral artery disease, systolic/diastolic blood pressure ≥ 140/90 mmHg, simvastatin equivalent dose of either 20 to 40 vs. 10 mg/day or > 40 mg vs. 10 mg/day and ezetimibe.

### Statistical analysis

To estimate the sample size we assumed a prevalence of lipid abnormalities between 20 and 60% and a design effect of 20% (variance inflation due to cluster sampling design). We calculated that, within this range, a sample size of 2100 would be sufficient to estimate the prevalence with a given precision of ±2.3% (range of 95% confidence interval 4.6%). Furthermore we determined that this size guaranteed enough information for estimating the prevalence in smaller subgroups (representing one quarter or more of the population) with a precision of ±4.7% (range of 95%-CI: 9.4%).

Data were entered into a central electronic (web-based) database housed and managed at the Institut für Herzinfarktforschung, Ludwigshafen, Germany, after original data was first collected in paper CRF form. Real-time quality control (internal logic checks) occurred during data entry.

Continuous variables were presented as means with standard deviations or medians with 25^th^ and 75^th^ percentiles (interquartile range [IQR]), as appropriate. Categorical variables were reported as absolute numbers and percentages. Kernel density estimation was used to estimate the distribution of total cholesterol, LDL-C, HDL-C, and triglyceride levels.

Kernel density estimation was used to analyze the distribution of total cholesterol, LDL-C, HDL-C, and triglyceride levels. The value of a kernel density and its slope at the lipid value equal to the ESC goal provides a crude indicator of the change in the proportions of patients meeting the goal from an improvement or deterioration in lipid level by a small amount starting from the ESC goal and thus provides a sensitivity analysis for either changes in the ESC goals or changes in lipid levels for people whose levels are near the goals. For each kernel density, the mode of the kernel density is in the region that meets the goal, but quite close to the cut point, so that deterioration in levels (or raising the goal) of any of the lipids local to the cut point would reduce the proportion meeting goal by a relatively large and increasing amount as successive changes in the lipid level approached the mode. Given that the mode for each meets the goal, an improvement in lipid levels that are local to the cut point that represents the goal have necessarily smaller effects than a deterioration in lipid levels.

Multiple logistic regression analysis with stepwise backward selection (α = 0.05) was performed to detect factors independently associated with LDL-C, HDL-C, and triglyceride abnormalities, with variables including patient characteristics, risk categories, medical history, statin use, and physician’s specialty. All factors were subjected to the stepwise backward selection procedure. The variables were maintained in the model if p<0.05 (Wald test). All models were not overfitted [19].

All statistical comparisons were two-tailed, and a p < 0.05 was considered significant. Patients who did not have the appropriate lipid parameters were not included in the lipid analyses. All analyses were performed using SAS v9.1 (SAS Institute Inc., USA).

## Results

### Patient characteristics

Patient characteristics, CVD risk categories and lipid parameters are presented in Table **1**. The mean age of patients was 59.4 years (men 58.9 years, women 60.3 years) and 87.7% were Arabic (men 86.3%, women 90.3%). As assessed using the 2011 ESC guidelines, most patients (82.6%) were at very high risk of cardiovascular complications (defined as having CVD, diabetes, and/or an ESC Systematic Coronary Risk Evaluation (SCORE) risk of ≥ 10%), with 85.0% of men and 77.7% of women defined as very high-risk. A substantially higher percentage of men *vs.* women had a diagnosis of CVD (60.2 vs. 36.5%, respectively). Current tobacco smoking was more prevalent in men (22%) than women (9.8%), while obesity and metabolic syndrome were more frequent in women (59.0 vs. 51.4%, respectively, and 81.9 and 72.7%, respectively). The prevalence of diabetes mellitus was similar in men (66.1%) and women (68.6%), and 96.6% of all cases of diabetes mellitus were type 2.

**Table 1:** Patient characteristics, risk categories and lipid parameters.

	**All patients** (n = 2,182)*	**Men** (n = 1,456)	**Women** (n = 724)
Age (years) [mean ± SD]	59.4 ± 9.4	58.9 ± 9.4	60.3 ± 9.4
Arabic (%)	87.7	86.3	90.5
Family history of premature CHD (%)	28.5	29.1	27.5
Current Smokers (%)	18.0	22.0	9.8
Hypertension (%)	78.1	76.8	80.8
Systolic BP (mmHg) [mean ± SD]	133.8 ± 15.8	133.5 ± 15.5	134.4 ± 16.4
Diastolic BP (mmHg) [mean ± SD]	80.2 ± 12.2	80.4 ± 12.5	79.8 ± 11.5
Waist circumference (cm) [mean ± SD]	101.9 ± 12.8	103.6 ± 11.9	98.2 ± 13.8
BMI (kg/m^2^) [mean ± SD]	30.6 ± 5.2	30.2 ± 4.7	31.4 ± 6.0
BMI ≥ 30 kg/m^2^ (%)	53.9	51.4	59.0
CVD (%)	52.3	60.2	36.5
Diabetes mellitus (%)	66.9	66.1	68.6
Diabetes mellitus Type 1 (%)	3.4	2.9	4.4
Diabetes mellitus Type 2 (%)	96.6	97.2	95.6
Metabolic Syndrome (IDF) (%)	75.7	72.7	81.9
ESC risk level (2011)			
Very high risk patient (%)	82.6	85.0	77.7
High risk patient (%)	2.7	2.9	2.4
Moderate risk patient (%)	10.3	10.8	9.3
Low risk patient (%)	4.4	1.3	10.7
Lipids			
LDL-C (mmol/L) [mean ± SD]	2.4 ± 0.9	2.4 ± 0.9	2.4 ± 0.9
HDL-C (mmol/L) [mean ± SD]	1.1 ± 0.4	1.0 ± 0.3	1.2 ± 0.4
Total cholesterol (mmol/L) [mean ± SD]	4.3 ± 1.3	4.2 ± 1.3	4.6 ± 1.2
Triglycerides (mmol/L) [median (IQR)]	1.6 (1.0-2.3)	1.6 (1.0-2.3)	1.7 (1.1-2.3)
Blood glucose			
Fasting plasma glucose (mmol/L)	6.6 (5.2-9.6)	6.7 (5.2-9.8)	6.5 (5.1-9.3)
HbA1c [%] in diabetic patients	7.9 (6.8-8.7)	7.9 (6.9-8.6)	7.8 (6.8-8.7)

Abbreviations: CHD, coronary heart disease; BP, blood pressure; BMI, Body mass index; CVD, cardiovascular disease; DM, diabetes mellitus; IDF, International Diabetes Federation; * for two patients information on gender was not available

### Lipid-modifying treatments and statin potency

**Figure 1:**
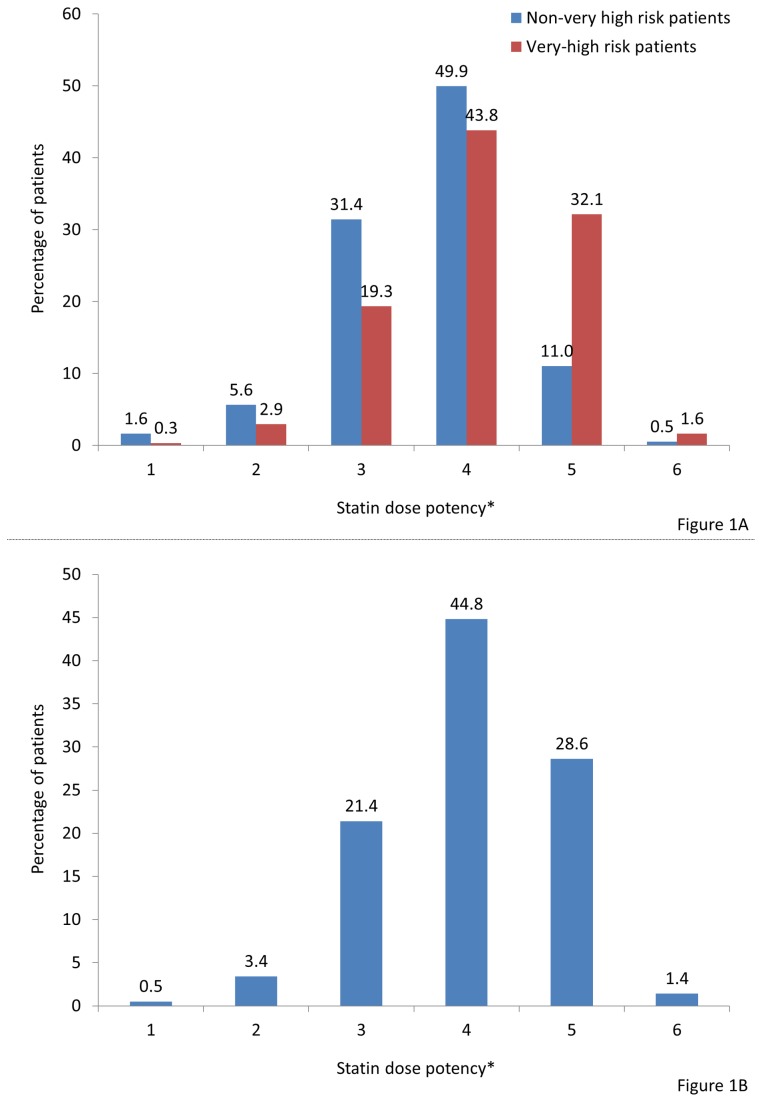
Statin dose potency according to patients' risk status (a) and overall (b) calculated according to [16,17]. * Statin dose potency 1 is equivalent to Simvastatin 5 mg/day, potency 2 is equivalent to Simvastatin 10 mg/day, potency 3 is equivalent to Simvastatin 20 mg/day , potency 4 is equivalent to Simvastatin 40 mg/day, potency 5 is equivalent to Simvastatin 80 mg/day, and potency 6 is equivalent to Simvastatin ≥160 mg/day.

Among patients (n = 2,165) for whom data on statin treatment were available, over half (54.5%) were receiving atorvastatin. Simvastatin and rosuvastatin were being prescribed to 26.8 and 14.7% of patients, respectively, and fluvastatin, pravastatin and lovastatin to 2.3, 1.1 and 0.5% of patients, respectively. Approximately one-quarter (26.7%) of patients (n = 2,180) for whom data were available were receiving other lipid-lowering treatments as follows: ezetimibe, either alone (15.6%) or in combination with a statin (2.0%), fibrates (13.7%), nicotinic acid (0.2%), bile acid sequestrant (0.1%). The most frequent statin dose potency was 4 for both very high-risk patients (43.8%) and non-very high-risk patients (49.9%). Approximately one-third of very high-risk patients (32.1%) were receiving a dose potency of 5 and approximately one-third of non-very high-risk patients (31.4%) were receiving a dose potency of 3 (Figures **1a and 1b**).

### Lipid abnormalities

Table **2** describes lipid profile in all patients with any data available (lower panel) and in patients for which all three values (LDL-C, HDL-C and TG) were available (total lipid profile). In patients with a total lipid profile the most prevalent lipid abnormality was LDL-C not at target level, which was observed in 61.8% of patients with a total lipid profile (n = 2,009), and 61.5% of all patients (n = 2,181).

**Table 2:** Lipid abnormalities according to ESC guidelines (2011).

**Patients with total lipid profile**	**All patients** (n = 2,009)	**Very high** risk* (n = 1,649)	**High Risk** (n = 58)	**Moderate risk** (n = 214)	**Low Risk** (n = 88)
LDL-C not at target [%]^†^	61.8	69.5	56.9	29.0	*^Δ^*
Low HDL-C (<1.0 [men]/1.2 [women] mmol/L) [%]	55.5	58.4	36.2	47.2	33.0
Elevated TG (>1.7 mmol/L) [%]	48.5	51.1	37.9	36.4	37.5
**All patients**	(n = 2,181)	(n = 1,801)	(n = 59)	(n = 224)	(n= 97)
LDL-C not at target [%]**^†,‡^**	61.5	69.5	56.9	29.0	*^Δ^*
Low HDL-C (<1.0 [men]/1.2 [women] mmol/L) [%]**^‡^**	56.8	59.8	37.3	47.8	33.7
Elevated TG (>1.7 mmol/L) [%]**^*§*^**	46.7	48.6	37.3	37.5	39.6

* Very high risk = CVD, Diabetes, and/or SCORE risk ≥10% (chronic kidney disease was not documented in DYSIS); ^†^ LDL ≥3.0 mmol/L in patients with SCORE risk 1-4%, LDL ≥ 2.5 mmol/L in patients with SCORE risk 5-9%, LDL ≥ 1.8 mmol/L in patients with CVD, DM, and/or SCORE risk ≥10%; data on 2,020 patients were available for “all patients”, ‡ Data on 2,165 patients were available, § Data on 2,166 patients were available; ^Δ^ in the ESC 2011 guidelines, no LDL-C goal was specified for the low risk group

**Figure 2:**
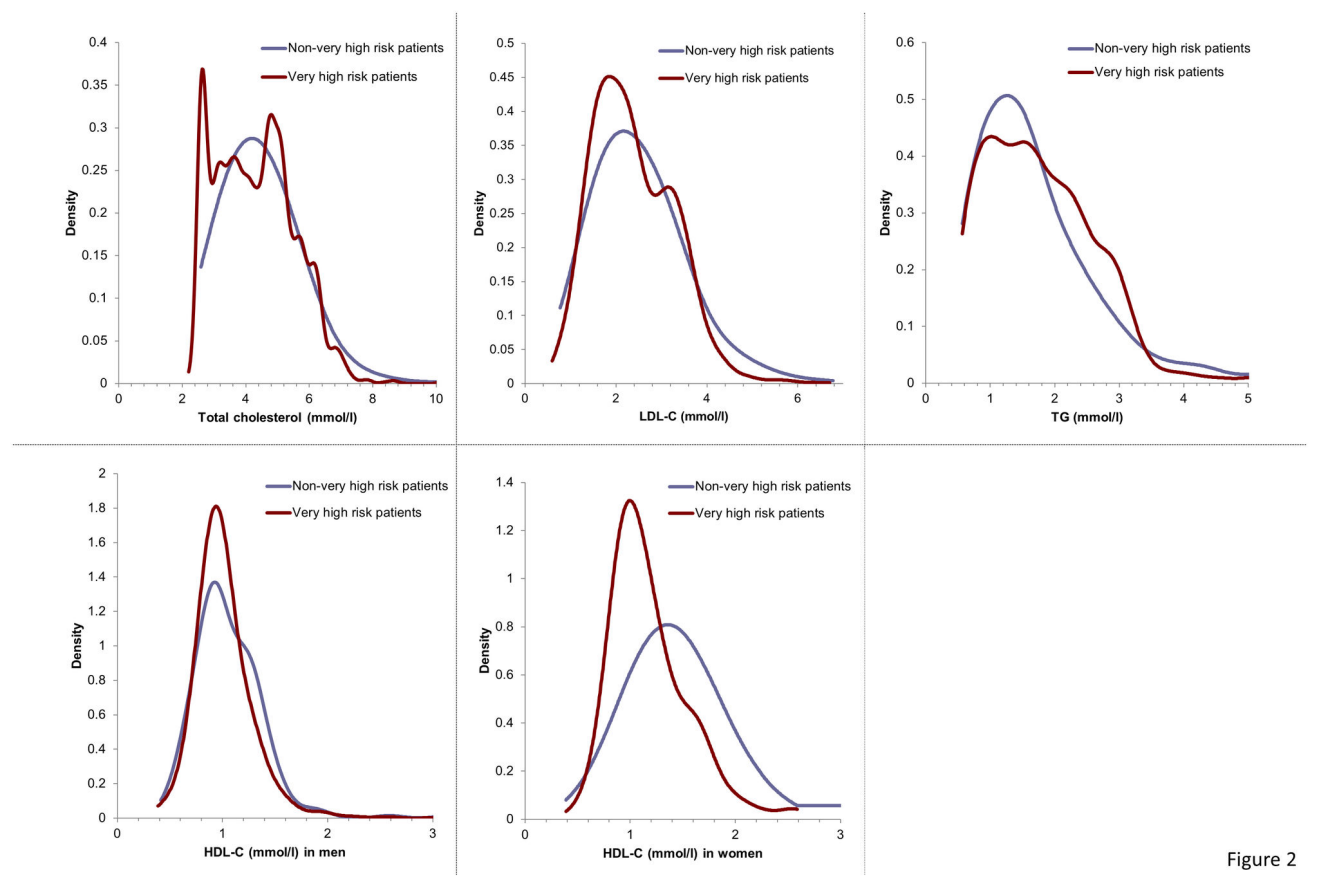
Kernel density curves of lipids. Vertical lines mark the cut point of ESC guidelines (2011).

Sub-analysis by CVD risk status for patients with a total lipid profile demonstrated that LDL-C levels were not at target in 69.5, 56.9, 29.0 and 0.0% of very high-, high-, moderate- and low-risk patients, respectively. Low HDL-C levels were present in 55.5% of patients with a total lipid profile, respectively, and in 58.4, 36.2, 47.2 and 33.0% of patients with very high, high, moderate and low risk, respectively. The prevalence of elevated triglyceride levels was similar to that of low HDL-C levels, detected in 48.5% of patients with a total lipid profile and in 51.1, 37.9, 36.4 and 37.5% of patients classified as very high, high, moderate and low risk, respectively. Sub-analyses of data on very high-risk patients (n = 753 with total lipid profile) with CVD and diabetes mellitus demonstrated that 74.4% had LDL-C levels ≥ 1.8 mmol/L, while 68.4% had low HDL-C levels, and 65.1% had elevated triglyceride levels (Table **3**). Kernel density curves illustrate that the proportion of women with low HDL-C levels, in particular for those at very high risk of cardiovascular events, was much higher than that of men (Figure **2**). 

**Table 3:** Lipid abnormalities according to ESC guidelines (2011) in very high risk patients.

**Patients with total lipid profile**	CVD + DM (n = 753)	**CVD** (**w/o DM**) (n = 280)	**DM** (**w/o CVD**) (n = 586)	**SCORE ≥ 10%** (n = 30)
LDL-C ≥ 1.8 mmol/L and LDL-reduction <50% [%]	74.4	63.9	65.4	80.0
Low HDL-C (<1.0 [men]/1.2 [women] mmol/L) [%]	68.4	52.9	48.3	56.7
Elevated TG (>1.7 mmol/L) [%]	65.1	38.2	39.1	53.3
**All patients**	(n = 829)	(n = 312)	(n = 629)	(n= 30)
LDL-C ≥ 1.8 mmol/L and LDL-reduction <50% [%]*	74.4	63.9	65.4	80.0
Low HDL-C (<1.0 [men]/1.2 [women] mmol/L) [%]†‡	69.4	54.8	50.0	56.7
Elevated TG (>1.7 mmol/L) [%]‡	61.1	35.3	38.7	53.3

* Data on 1,650 patients were available, † Data on 1,787 patients were available, ‡ Data on 1,786 patients were available

**Figure 3:**
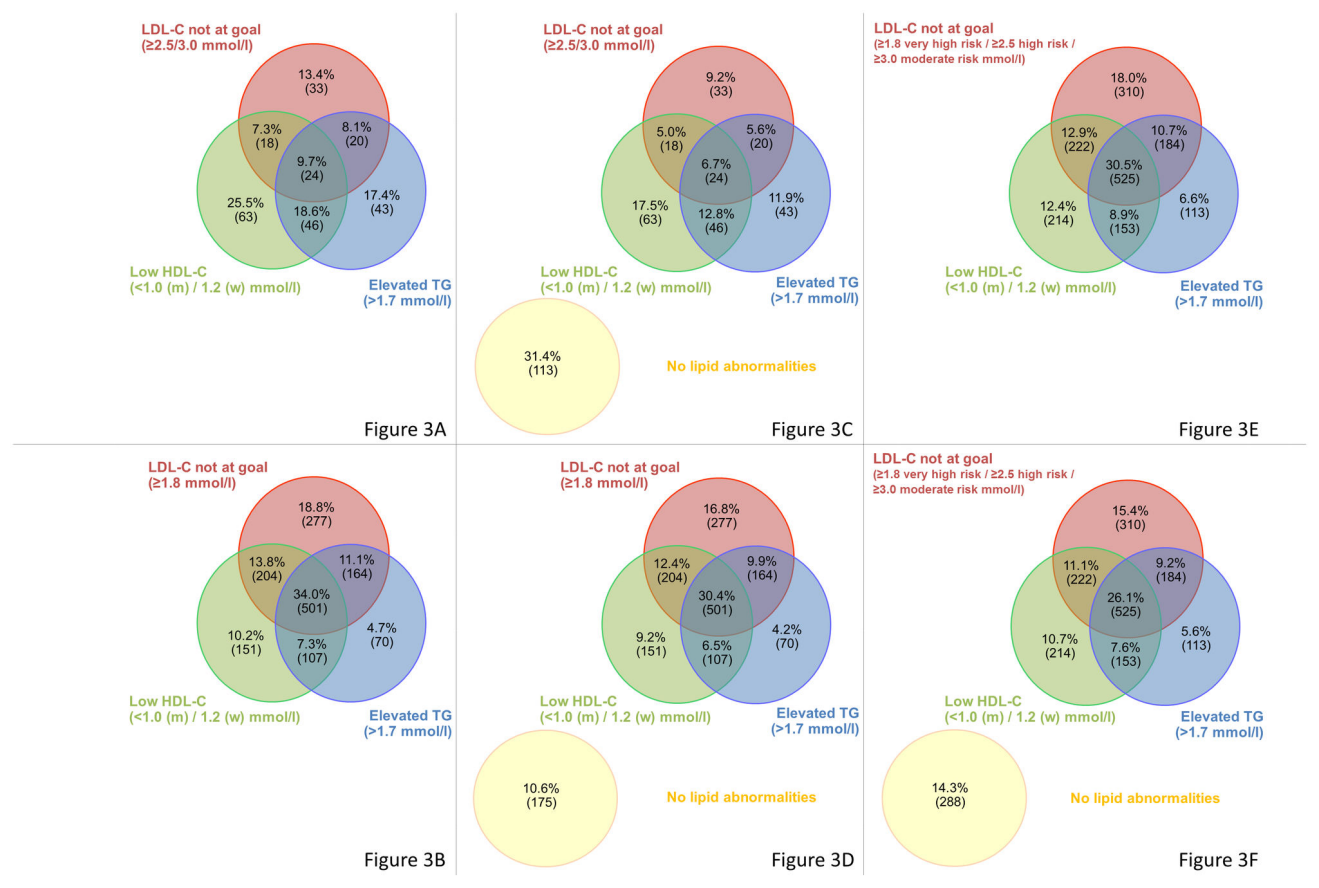
Distribution of single and multiple combined lipid abnormalities. 3a. Non-very high risk patients (ESC 2011, SCORE <10%) with at least one lipid abnormality. 3B. Very high risk patients (ESC 2011, CVD, diabetes and/or ESC-SCORE ≥10%) with at least one lipid abnormality. 3C. Non-very high risk patients (ESC 2011, SCORE <10%). 3D. Very high risk patients (ESC 2011, CVD, diabetes and/or ESC-SCORE ≥10%). 3E. Patients with at least one lipid abnormality. 3F. Patients with total lipid profile.

The distribution of single and multiple combined lipid abnormalities in all patients with a total lipid profile, patients who are not very high risk and very high-risk patients is presented in Figures **3a, 3b** ****and **3c**. In all patients with a total lipid profile, 14.3% had normal lipid levels and 26.1% had all three lipid abnormalities. In patients not at very high risk, the most common lipid abnormality was low HDL-C levels (41.9%), followed by elevated triglyceride (37.0%) and LDL-C levels (26.5%). Lipid levels were within target range in 31.4% of patients not at very high risk and all three lipid abnormalities were observed in 6.7% of this patient group. For very high-risk patients, the most frequent abnormality was LDL-C levels not at target (69.5%), followed by low HDL-C (58.5%) and elevated triglyceride levels (51.1%). Only 10.6% of very-high risk patients had lipid levels within target range and 30.4% of these patients exhibited all three lipid abnormalities. Among patients with at least one lipid abnormality, the most common lipid disorders were LDL-C levels not at target in all patients (72.1%) and very-high risk patients (77.7%), and low HDL-C levels in patients not at very high risk (61.1%; Figures **3d, 3e** ****and **3f**).

### Factors associated with dyslipidemia

Table **4** displays the results of multivariate logistic regression analysis, which allowed the identification of several variables being associated with abnormal LDL-C, HDL-C and triglyceride levels. LDL-C levels not at target were positively associated with a family history of premature CHD, current smoking, sedentary lifestyle, diabetes mellitus, ischemic heart disease, cerebrovascular disease, high blood pressure and a simvastatin equivalent dose of > 40 mg/day (versus 10 mg/day). They were negatively associated with age ≥ 70 years and alcohol consumption > 2 units/week. Variables positively associated with low HDL-C levels included current tobacco smoker, sedentary lifestyle, obesity, diabetes mellitus, ischemic heart disease, cerebrovascular disease, a simvastatin equivalent dose of > 40 mg/day (versus 10 mg/day), and ezetimibe. Female gender had a negative association with low HDL-C levels. 

**Table 4:** Factors independently associated with for LDL-C, HDL-C, and triglyceride (TG) abnormalities: results of multivariable logistic regression (OR, 95%CI).

	LDL-C not at target* (≥1.8/2.5/3.0 mmol/L)	Low HDL-C* (<1.0 (m)/1.2 (w) mmol/L)	Elevated TG* (>1.7 mmol/L)	LDL-C not at target and low HDL-C & elev. TG*
Age ≥70 years	0.67 (0.51-0.90)	n.s.	0.61 (0.47-0.80)	0.70 (0.50-0.97)
Female	n.s.	0.80 (0.65-0.98)	n.s.	1.37 (1.05-1.79)
Family Hx of premature CHD	1.31 (1.03-1.66)	n.s.	1.37 (1.10-1.70)	1.51 (1.17-1.97)
Current smoker	1.70 (1.28-2.25)	2.16 (1.67-2.81)	n.s.	2.33 (1.71-3.17)
Sedentary lifestyle	1.32 (1.05-1.67)	1.52 (1.23-1.88)	1.83 (1.46-2.29)	3.58 (2.47-5.18)
Alcohol consumption > 2 units/week	0.28 (0.09-0.92)	n.s.	3.26 (1.28-8.27)	n.s.
BMI ≥30 kg/m^2^ (obesity)	n.s.	1.21 (1.00-1.46)	1.35 (1.11-1.64)	1.68 (1.27-2.21)
WC >102 (m) / >88 cm (w)	n.s.	n.s.	n.s.	0.72 (0.55-0.96)
Hypertension	n.s.	n.s.	1.53 (1.19-1.95)	2.04 (1.38-3.03)
Diabetes mellitus	2.81 (2.28-3.48)	1.44 (1.18-1.76)	1.34 (1.08-1.65)	2.56 (1.89-3.45)
Ischemic heart disease	1.66 (1.34-2.07)	1.38 (1.13-1.69)	n.s.	1.67 (1.28-2.18)
Cerebrovascular disease	2.20 (1.41-3.42)	1.98 (1.36-2.88)	1.84 (1.27-2.66)	1.57 (1.09-2.28)
BP ≥140/90 mmHG (systolic/diastolic)	1.64 (1.33-2.01)	n.s.	1.79 (1.47-2.19)	1.97 (1.53-2.53)
Simvastatin 10 mg/day	1.0 (reference)	1.0 (reference)	1.0 (reference)	1.0 (reference)
20-40 vs. 10 mg/day	n.s.	n.s.	0.67 (0.55-0.82)	n.s.
> 40 vs. 10 mg/day	1.56 (1.23-1.99)	1.48 (1.19-1.84)	n.s.	1.80 (1.40-2.32)
Ezetimibe	n.s.	1.95 (1.49-2.56)	2.85 (2.18-3.71)	2.48 (1.88-3.28)

^*^ Models contained the following variables: age, sex, 1^st^ grade family history of premature CVD, current smoker, sedentary lifestyle, alcohol consumption > 2units/week, BMI ≥30 kg/m^2^ (obesity), waist circumference >102 cm in men / >88 cm in women, hypertension, diabetes mellitus, ischemic heart disease, cerebrovascular disease, heart failure, peripheral artery disease, RR ≥140/90 mmHg (systolic/diastolic), 20-40 vs. 10 mg/day Simvastatin equivalent, ≥ 80 vs. 10 mg/day Simvastatin equivalent, ezetimibe

Backward selection (alpha=0.05) was done

Abbreviations: m = men, w = women, BP = blood pressure, n.s. = not significant (p > 0.05), OR = odds ratio, CI = confidence interval

Elevated triglyceride levels were associated with family history of CHD, sedentary lifestyle, alcohol consumption > 2 units/week, obesity, hypertension, diabetes mellitus, cerebrovascular disease, and blood pressure ≥ 140/90 mmHg. Negative associations were seen with age ≥ 70 years, a simvastatin equivalent dose of 20 to 40 mg vs. 10 mg/day and ezetimibe. Most variables were independently associated with the presence of all three lipid abnormalities with the exception of alcohol consumption, heart failure, PAD, and simvastatin equivalent doses of 20 to 40 mg vs. 10 mg/day.

## Discussion

Results from the DYSIS-Middle East study, in which more than three-quarters of patients were classified as very high-risk, demonstrated that the most prevalent lipid abnormality, despite chronic statin treatment, was elevated LDL-C levels. Goal LDL-C levels were not attained in 61.8% of all patients and in 69.5% of very high-risk patients. Low HDL-C and elevated triglyceride levels were both observed in approximately 50% of all patients (7.6% had low HDL-C and elevated TG at a normal LDL-C). Overall, dyslipidemia was present in 85.7% of all patients. An analysis of pooled data from the Canadian and European cohorts of the DYSIS study demonstrated that 48.2% of patients had LDL-C levels not at target [15]. Thus, in the DYSIS-Middle East study, the proportion of patients with LDL-C levels not at target was higher than that in the overall DYSIS study.

### Variables associated with lipid abnormalities

In multivariable logistic regression analyses, among the strongest variables associated with lipid abnormalities were current tobacco smoking as well as a sedentary lifestyle. Other factors associated with LDL-C not at target included diabetes mellitus, ischemic heart disease and blood pressure ≥ 140/90 mmHg. Surprising results of DYSIS Middle East were the positive association of high dose statin treatment and ezetimibe with poor lipid control. Because of this we explored potential explanations for this unexpected findings and found the following: 1) The control group against which ORs were calculated (5-10 mg/day) was rather small (n=83), resulting in considerable uncertainty with respect to control rates in this group. 2) Patients in the high dose statin group had considerably increased baseline LDL-C values (237.3±116.8 mg/dl vs. 130.1±59.7 in those receiving the lowest dose). 3) The majority of patients in the high dose statin group (93.4%) were classified as being at very high risk prompting very low treatment targets of < 1.8 mmol/l). These results taken together may have resulted our finding, which is reflected by the results of the European analysis in 22063 patients where high dose statins and ezetimibe were associated with improved control rates [15].

### Improving lipid level control

Strategies for improving lipid level control, in particular intensive lipid-lowering treatment, have been investigated in several large, non-interventional studies. For example, the Austrian Cholesterol screening and Treatment (ACT) II study evaluated the effect of intensifying lipid-lowering therapy in high-risk patients with elevated LDL-C levels despite statin treatment. Recently published data from this study demonstrate that after 12 months of intensified therapy, 40.3% of patients met their LDL-C goals, with a decline in LDL-C levels from baseline of 31.3% and an increase in HDL-C levels of 11.9%. The most commonly prescribed treatment was a fixed-dose combination of simvastatin and ezetimibe (73% of patients) [20]. These data draw attention to the limitations of currently available therapeutic agents and suggest that high dose levels of statins and combination therapies should be used more widely. This idea is supported by pooled data from the European and Canadian cohorts of the DYSIS study - key variables associated with attaining target LDL-C levels were: higher statin dose levels, specialist treatment, or combined lipid-lowering therapy [18]. Indeed, the implementation of intensive statin therapy and combinations of lipid-lowering therapies for patients who don’t reach target lipid levels is expected to increase [21,22].

Given the strong association in the DYSIS-Middle East Study between both single and multiple lipid abnormalities, and either tobacco smoking or a sedentary lifestyle, changes in lifestyle choices are also vital for normalization of lipid levels and reduction in cardiovascular risk. In Saudi Arabia, UAE and Lebanon, it is estimated that 66.8, 58 and 47% of the respective populations have a sedentary lifestyle, and in Saudi Arabia, 22.6% of adults are tobacco smokers [23]. Furthermore, many regions in the Middle East require improved surveillance, awareness and characterization of cardiovascular risk factors [24,25].

### Limitations

The design of the DYSIS study has several limitations. As a cross-sectional study, data were only taken from a single time-point and long-term evaluation of cardiovascular risk was not possible. In the majority however we made use of the CardioChek device, which allows direct LDL-C determinations at the time of the evaluation. Furthermore it is possible that patients’ treatment regimens could have changed. However, this reflects the real-life clinical situation. Thirdly, statin treatment was a pre-requisite for inclusion, which could have introduced self-selection bias, inferring that this study may overestimate the use of statins.

### Clinical implications

The prevalence of lipid abnormalities in the setting of chronic statin treatment has been assessed in numerous cross-sectional studies [13,20,26]. In contrast to the present study, many of these studies have focused on LDL-C levels alone, specific populations of patients (e.g., those with or without established coronary heart disease), or included patients both on and off lipid-lowering treatment. Thus, DYSIS-Middle East, being the largest survey on dyslipidemia conducted to date, provides an up-to-date perspective on statin treatment in clinical practice, and highlights the need for more effective treatment regimens and the development of novel lipid-lowering therapies, particularly in light of the high prevalence of very high-risk patients.

## Conclusions

DYSIS-Middle East demonstrates that despite statin therapy, a high proportion of patients fail to meet lipid targets and many have a very high risk of CVD. Further studies should be conducted with the aim of extending these results and providing a greater body of evidence on risk factors for CVD and treatment strategies in Middle Eastern patients.
